# The impact of the striped field mouse’s range expansion on communities of native small mammals

**DOI:** 10.1038/s41598-022-26919-z

**Published:** 2023-01-14

**Authors:** Filip Tulis, Michal Ševčík, Radoslava Jánošíková, Ivan Baláž, Michal Ambros, Lucia Zvaríková, Gyözö Horváth

**Affiliations:** 1 Department of Ecology and Environmental Science, Faculty of Natural Sciences and Informatics, Constantine the Philosopher University in Nitra, Tr. A. Hlinku 1, SK-949 74 Nitra, Slovak Republic; 2 State Nature Conservancy of SR, Administration of Protected Landscape Area Ponitrie, Samova 3, SK-949 01 Nitra, Slovak Republic; 3 Department of Ecology, Institute of Biology, Faculty of Sciences, University of Pécs, Ifjúság Útja 6, H-7624 Pécs, Hungary

**Keywords:** Biodiversity, Community ecology

## Abstract

Understanding species expansion as an element of the dispersal process is crucial to gaining a better comprehension of the functioning of the populations and the communities. Populations of the same species that are native in one area could be considered nonindigenous, naturalised or invasive somewhere else. The striped field mouse has been expanding its range in south-western Slovakia since 2010, although the origin of the spread has still not been clarified. In light of the striped field mouse’s life history, the recent range expansion is considered to be the expansion of a native species. This study analyses the impact of the striped field mouse's expansion on the native population and small mammal communities and confronts the documented stages of striped field mouse expansion with the stages of invasion biology. Our research replicates the design and compares results from past research of small mammals prior to this expansion at the same three study areas with the same 20 study sites and control sites. Several years after expansion, the striped field mouse has a 100% frequency of occurrence in all study sites and has become the dominant species in two of the study areas. The native community is significantly affected by the striped field mouse’s increasing dominance, specifically: (i) we found a re-ordering of the species rank, mainly in areas with higher dominance, and (ii) an initial positive impact on diversity and evenness during low dominance of the striped field mouse turned markedly negative after crossing the 25% dominance threshold. Results suggested that the variation in the striped field mouse’s dominance is affected by the northern direction of its spread. Our findings show that establishment in a new area, spread and impact on the native community are stages possibly shared by both invasive and native species during their range expansion.

## Introduction

Expansion is a consequence of the dispersal process, and the dynamics of expansion shape species distribution and community composition^1,2^. In light of current global changes, understanding range expansion mechanisms is an increasingly important challenge for ecology and conservation. A growing number of species are expanding their distribution range—invasive species are rapidly spreading into new areas, and native species are following their shifting climatic envelope^2^—but the boundary between invasive and native range expansion is not always clear.

Native species occur within their natural ranges (past or present) and in their dispersal potential^3^. In contrast, non-native species have been human-mediated, either accidentally or deliberately, outside of their natural ranges, regardless of their eventual impact on native ecosystems^4^. While “non-native” can be neutral in terms of their effect on the environment, “alien” or “invasive species” represent those non-native species that have a demonstrable impact, significantly modifying or disrupting the ecosystems that they colonise and, thus, being characterised as invaders^5^. Invasive species must pass through four stages to become invasive: (1) transport, (2) establishment, (3) spread, and (4) impact^6^. Furthermore, becoming “invasive” (both widespread and locally dominant), is always the culmination of a process that climaxes in an increase in abundance^7^. Due to the lack of natural enemies and appropriate conditions in the territories that they have newly occupied, invaders can introduce new pathogens, increase competition for resources (the novel weapons hypothesis^6^) and, ultimately, reduce the occurrence of native species to a level that threatens the indigenous species with extinction^5^. The responses of native species to an invasion depend critically on invasive species’ abundance and trophic position. As the abundance of an invasive species increases, native population sizes decline nonlinearly by an average of 20%, and community diversity declines linearly by 25%, with significantly larger effects on species evenness and diversity than on species richness (reviewed by^9^).

In the Anthropocene, alien species are no longer the only category of biological organisms establishing themselves and rapidly spreading beyond their historical boundaries^10^. Changes in the distribution of native plants^11,12^, birds^13–15^ and even small mammals^16–18^ have become increasingly common in recent decades. Nackley et al.^10^ pointed out that invasions by native species are a global phenomenon, and the expansion of native organisms into adjacent communities can emulate functional and structural changes and impact the biodiversity associated with invasions by alien species. However, Tong et al.^19^ argued against treating the expansion of native species in the same way as the arrival and expansion of alien species. Expanding native species can move together with their associated organisms (e.g., herbivores or parasites) and, thus, do not benefit from an enemy release like invasive alien species, making the effects of range expansion on local communities typically more neutral and milder than those of alien species invasion. However, Thompson and Davis^20^ suggested that, with continual global changes in nutrients, climate and disturbance regimes, all species can be considered to be inhabiting novel environments, and, therefore, distinctions between native and non-native species are becoming even less ecologically meaningful. Since biological invasions are an extension of normal colonisation processes, the terminology used should reflect this fact and not be based on the species’ geographic origins^21–23^. Therefore, the impact of species on the environment should be principally assessed^24^.

The striped field mouse (*Apodemus agrarius*) is an epidemiologically significant small mammal species^25,26^ that is widely distributed over the entire Palaearctic region, from Central Europe to the Korean Peninsula and the Russian Far East^27^. It spread to Europe in the first half of the Holocene, together with a specific parasite, *Hystrichopsylla orientalis orientalis*, a flea subspecies^28^. While the striped field mouse retreated from parts of Western and Central Europe, the parasite has persisted and survived thereafter on other hosts until the present day^29^. Over the past decade, there have been several documented expansion waves of the striped field mouse’s range into the Russian Far East^30^, Central and Eastern Europe^31–33^ and directly into Slovakia^34^. However, paleontological findings^35,36^ and the subrecent diet of owls^37,38^ indicate the occurrence of the striped field mouse in parts of Central Europe that are either supposedly not colonised or have been already re-colonised by them. But recent evidence of the species' occurrence in Western^39,40^ and Central Europe^32,41,42^ points to the considerable dynamics at the edge of their distribution, where their spread into new territory was subsequently followed by a retreat from the colonised land over a period of several decades.

**Figure 1:**
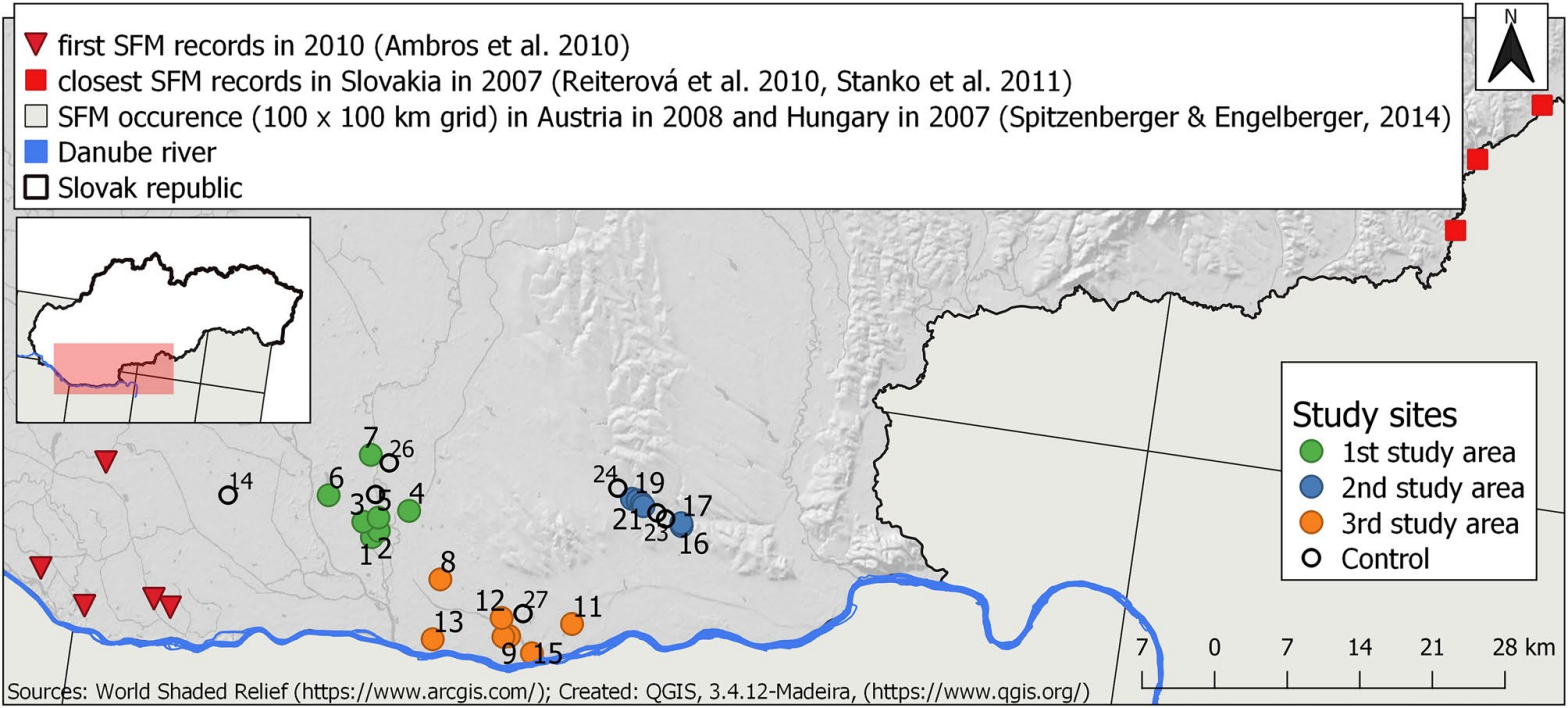
Study sites distributed in three study areas (green, blue, orange) and control sites (black circle), with the first striped field mouse (SFM) records in 2010 in south-western Slovakia (red triangles) and the closest known populations of striped field mice in the south of Slovakia in 2007 (red squares) and in 100 × 100 km grids in Austria and Hungary in 2013.

In 2010, the occurrence of the striped field mouse was first documented in our study region^8^; previous studies of small mammals and the diet of owls in this area^43–52^ have been made for more than a century, but no evidence of the striped field mouse was recorded. The closest known populations of the species in Slovakia were approximately 120 km east of the study area from 2006 to 2007^53,54^. Furthermore, closer new populations at another location south of our study area near the Danube river were confirmed earlier in Austria and Hungary^55^ (Fig. 1). Ambros et al.^8^ notes that no subfossil or fossil evidence of the striped field mouse has been found in the study area before. Authors hypothesised about the possible transport of striped field mice with the transit of municipal and industrial waste as a possible explanation for their presence. The fact is that in a few years, the species had expanded to a large part of south-western Slovakia^56,57^, and the expansion is continuing^58^. The enormous scale and speed of their expansion do not support the hypothesis of anthropochory, and indications and evidence supporting this supposition are lacking. However, the origin of this spreading population still remains unexplained. The change in the mean annual and summer temperatures in Central Europe has been identified as a possible driver for the current oscillation in the species’ western limit of distribution in Austria, Czechia, Hungary and Slovakia^55^. On the other hand, deforestation, conversion of steppe into farmland, and the construction of irrigation channels have been identified as drivers of striped field mouse range expansion in North Asia^30^.

Factors including the theoretical framework described above, current knowledge, the long-term presence of the striped field mouse's flea documented in the study area before the onset of the current spread^59–61^, the still unexplained origin of the spreading population and, most importantly, the fact that the fundamental and first stage of the invasion process is the transport of a non-indigenous species^6^, lead us to conclude that the current expansion of the striped field mouse in the study region is a range expansion of a natural species. Despite several documented changes in the range of the striped field mouse in Central and Eastern Europe, the impact of this expansion on native small mammals has never been studied. This study replicates intensive and comprehensive research conducted in the past on small mammals in the study area before the striped field mouse’s expansion, and it analyses the impact of the expansion on their native populations and communities.

This study aims to (i) analyse the impact of the striped field mouse's expansion on the native population and small mammal communities, and (ii) confront documented stages of the striped field mouse’s expansion with stages of invasion biology.

## Material and methods

### Study area

The research was conducted at 20 study sites divided into three study areas: the first study area was located along the original flow of the Nitra River and consisted of seven study sites; the second study area consisted of six study sites along the Paríž channel; the third area was along the original flow of the Žitava River, and it had seven study sites (Fig. 1). Both rivers in the study area originally meandered before they were straightened in the early twentieth century and channels dug to drain the floodplain, leaving behind distributaries and oxbow lakes today. All the study sites were situated adjacent to wetland habitats. The traps themselves were set at the edge of wetland habitats with agricultural habitats. The entire study area is classified as the northern Pannonian Plain and Hronská pahorkatina highlands. The mean annual temperature is 9.9 °C, with January the coldest month (mean temperature − 2.1 °C) and July the warmest (+ 20.5 °C). The study area is among the driest parts of Slovakia^62^, with an average annual precipitation of between 550 and 600 mm^63^.

### Study design

Before-After Control-Impact (BACI) design was used to identify the impact of striped field mouse expansion to native communities. BACI designs are commonly used to evaluate impacts from natural perturbations^64^ and management actions^65–67^, as well as for a wide variety of smaller-scale field experiments^68^. Impact/treatment effects for BACI designs are often estimated with a significant treatment (Control–Impact) × time (Before–After) interaction, indicating that the experimental treatment has truly had an effect on the impact sites. Considering the interaction term in this manner allows treatment impacts to be distinguished from the background time effects shared by all sites, and from the background differences between control and treatment sites^69^. Therefore, we used three types of data:

### 1st trapping period: small mammal native community data

Data about the original communities of small mammals before the expansion of the striped field mouse’s range came from research conducted in 1981–1983, 1990 and 2000–2006. The data consist of 772 individuals from 10 species that were caught at 20 study sites (during 20 sessions) divided into three study areas during different seasons (Supplementary S1). The plotted rarefaction curve shows species diversity accumulating in all study areas (Supplementary S2).

### 2nd trapping period: Recent small mammal community data

Data were gathered in 2019–2020 after the striped field mouse’s expansion with the same methodology, same catching efforts at the same study sites and the same seasonality as data from the 1^st^ trapping period (Supplementary S1). The data consist of 868 small mammals from 11 species which were captured at 20 locations (during 20 sessions). The communities indicated an increase and saturation of species diversity at all three study areas after the striped field mouse’s expansion (Supplementary S2).

### Control sampling

Data were gathered during two time periods, the 1st and 2nd control periods, at seven control sites. In both periods, small mammals were trapped at the time before the confirmed occurrence of striped field mouse at the control sites (Table 1).

**Table 1:** List of trapping at control sites with the year of first striped field mouse capture.

Control site no	1st control period	2nd control period	Season	Time gap (years)	First striped field mouse trapping (year)
14	2001	2011	spring	6 years	2013*
22	1981	2001	spring	9 years	2012**
23	1981	2001	spring	10 years	2012**
24	1981	2001	summer	8 years	2012**
25	2005	2011	summer	20 years	2013***
26	2002	2011	summer	20 years	2013***
27	1982	1990	autumn	20 years	2014****

No striped field mouse was captured: *in 2010, 2012; **in 2002, 2010 ***2012; ****in 2004, 2005.

Six control sites (no. 22–27) were situated in the study areas, and one (no. 14) was outside of all the study areas (Fig. 1).

This design was used due to the lack of localities (with the same or similar environmental conditions and the same native species pool) in which the striped field mice was absent at the time of this study 2019–2020^58^. Our research thus does not have the classical concept of BACI design (after-control in a similar time as impact-control). This type of design can still help us compare changes in the composition of paired stable and disturbed communities over time.

The average time interval between trapping periods was 13.3 years ± 5.9 SD. The criteria of the control sites were the same: season, methodology of trapping, and species diversity accumulation (Supplementary S2). The data consist of 514 small mammals from 10 species, which were used in two control periods: 203 in the 1st control period and 311 in the 2^nd^ control period (Supplementary S1).

At all control sites, more trapping sessions were carried out which showed the absence of striped field mice (none were captured). These negative sessions did not meet the methodological criteria (season, types of traps, sampling, etc.) and could not be included in further analysis (Supplementary S1). However, these data supported the later colonisation of the control sites and the absence of striped mice at the control sites during the control captures (Table 1).

### Small mammal trapping

Small mammals were captured in 50 box-type single live-traps (75 × 95 × 180 mm)^70^ placed in a line (with a 10 m gap) and left exposed over two or three nights at each study site. The traps were baited with a mixture of cereals, apples and meal worms. Traps were checked twice a day, in the morning and evening. All the individuals caught in 1st trapping period were then euthanised with isoflurane inhalation or CO_2_ (lethal dose in a closed chamber). During the 2nd trapping period the following species, which together represent only 1.37% of the total captured specimens, were trapped, sexed, aged, marked and then released: pannonian root vole (*Alexandromys oeconomus mehelyi*), steppe mouse (*Mus spicilegus*), lesser white-toothed shrew (*Crocidura suaveoelens*), bi-coloured white-toothed shrew (*Crocidura leucodon*) and Miller´s water shrew (*Neomys anomalus*). Only their first capture (without recaptures) during the session was used in the following analyses. Other trapped small mammals were euthanised in situ with isoflurane inhalation (lethal dose in a closed chamber), in compliance with Slovakian national law (https://www.epi.sk/zz/2012-377), placed separately in a secure container and transported within two hours to a laboratory where they were identified using morphological techniques, sexed, aged, and weighed. The tissues (lung, liver, kidney, heart, stomach and part of the tail) were collected and stored at -80ºC for subsequent virological, parasitological and molecular analysis^71–73^.

### Data exploration and statistical analysis

Species with minimal occurrence (< 3) in the whole dataset—namely, the steppe mouse (two occurrences), bi-coloured white-toothed shrew (two occurrences) and Miller’s water shrew (two occurrences)—were not included in the statistical analysis. Prior to analysis, data were explored for residual effects of different sampling efforts by rarefaction on basic Hill numbers; *q0* = richness, *q1* = exponential Shannon index, *q2* = inverse Simpson index (Supplementary S2).

### Community distribution and dynamics

To analyse the types of community distribution, we used rank abundance curves (RAC; also called as dominance–diversity curves) with all five models supported by the vegan R package (Broken stick, Preemption, Lognormal, Zipf and Mandelbrot). The best model for each study area and time period (before and after striped field mouse expansion) was determined by the Akaike information criterion (AIC) for the best description of the observed pattern. In general, for a smaller number of samples (sample size/number of estimated parameters < 40), it is recommended to use second-order AIC (AICc—corrected)^74^. Since the tool used (*vegan::radfit*) does not offer AICc, it was calculated manually for each model^75,76^. For a closer look at community changes before and after expansion, five aspects of rank abundance curves—richness, evenness, rank, species gain and species loss—were calculated.

### Community metric changes

Due to the slight uncertainty regarding the *q0* (species richness) of the third area shown during rarefaction analysis (Supplementary S2) and the general deficiency of *q0* in diversity analyses^77^, we decided to use the more common species–sensitive *q2* index for further analysis to meet our goal.

Based on the RAC results, we decided to include the Berger-Parker (*d*) dominance index to better measure differences in community evenness influenced by the presence of the striped field mouse. The Berger-Parker index has a close relationship with evenness and refers to disproportionately abundant species. While common indices—such as Shannon, inverse Simpson and evenness—increase with improving community composition, an increase in the *d* index indicates the instability of the community due to the most dominant species. Species evenness and dominance measures may also be related to the net primary productivity of a system, invasion susceptibility and local extinction patterns^78^.

Since the range of both indices did not include limit values, differences in indices between areas and time periods (before and after expansion) were tested with linear mixed models with Gaussian error distribution, where pairs of the same localities were used as random factors (paired). Due to singularity problems in restricted maximum-likelihood models (e.g., the variance of a random variable can be miscalculated), Bayesian linear mixed models were used. All models were fitted with the “Bayesian Regression Models using Stan” (*brms*) R package^79^ with four chains, where each chain had a warm-up of 2000 iterations and then 4000 samples. We confirmed algorithm convergence with visual checks and the Rhat statistic. We chose informative priors that promoted the shrinkage of effects towards zero, including *N(0, 2)* prior for *q2* and *N(0, 0.5)* prior for the *d* index. The importance of interaction was evaluated by comparing it to models without interaction, using leave-one-out cross-validation scores (LOOCV).

### Impact of striped field mouse’s dominance

The relationship between the dominance of the striped field mouse and *q2* and *d* was described using simple linear regression. To test the unimodal rather than the linear response, we used second-degree polynomials. Maximum (for *q2*) and minimum (for *d*) values of the fitted final polynomial model were computed with a golden section search, which is an optimisation technique for finding an extremum (minimum or maximum) of a unimodal function inside a specified interval.

### Community composition changes

The compositional community variation between the control and the study areas at different times was analysed using multivariate general linear models with negative-binomial error distribution and a log link function using the *mvabund* R package^80^. The significance of the individual factors was obtained by applying the PIT-trap “model-free bootstrap” sampling method^81^ to 999 permutations tested with likelihood-ratio test (LRT) statistics. For further multiple comparisons, post hoc analysis via a free step-down resampling procedure with Holm adjusted *p*-values was used.

To visualise the multivariate relationship, non-metric multidimensional scaling (NMDS) was used with the Bray Curtis dissimilarity index. Changes in community composition and the domination of the striped field mouse in the north-west direction were tested using penalised regression splines with a generalised additive model (GAM) with 10 dimensions and a thin plate regression spline smoothing basis, through the *vegan* package^82^(Oksanen et al., 2020). All analyses were performed in the R environment^83^.

### Ethics statement

All methods were performed in accordance with the relevant guidelines and regulations of the institution or practice at which the studies were conducted. The research and methods were approved by the Ethics Committee of the Constantine the Philosopher University in Nitra. All trapping and subsequent handling of the small mammals was performed with the approval of the Ministry of Environment of the Slovak Republic in accordance with permission no. 4850/2019–6.3, dated 25th April 2019. The research was conducted by virtue of the appointment of Dr. Michal Ambros (employed by the State Nature Protection of the Slovak Republic) as mapping coordinator for important European small mammal species. This study follows the recommendations in the Animal Research: Reporting of In Vivo Experiments (ARRIVE) guidelines 2.0^84^.

## Results

**Figure 2:**
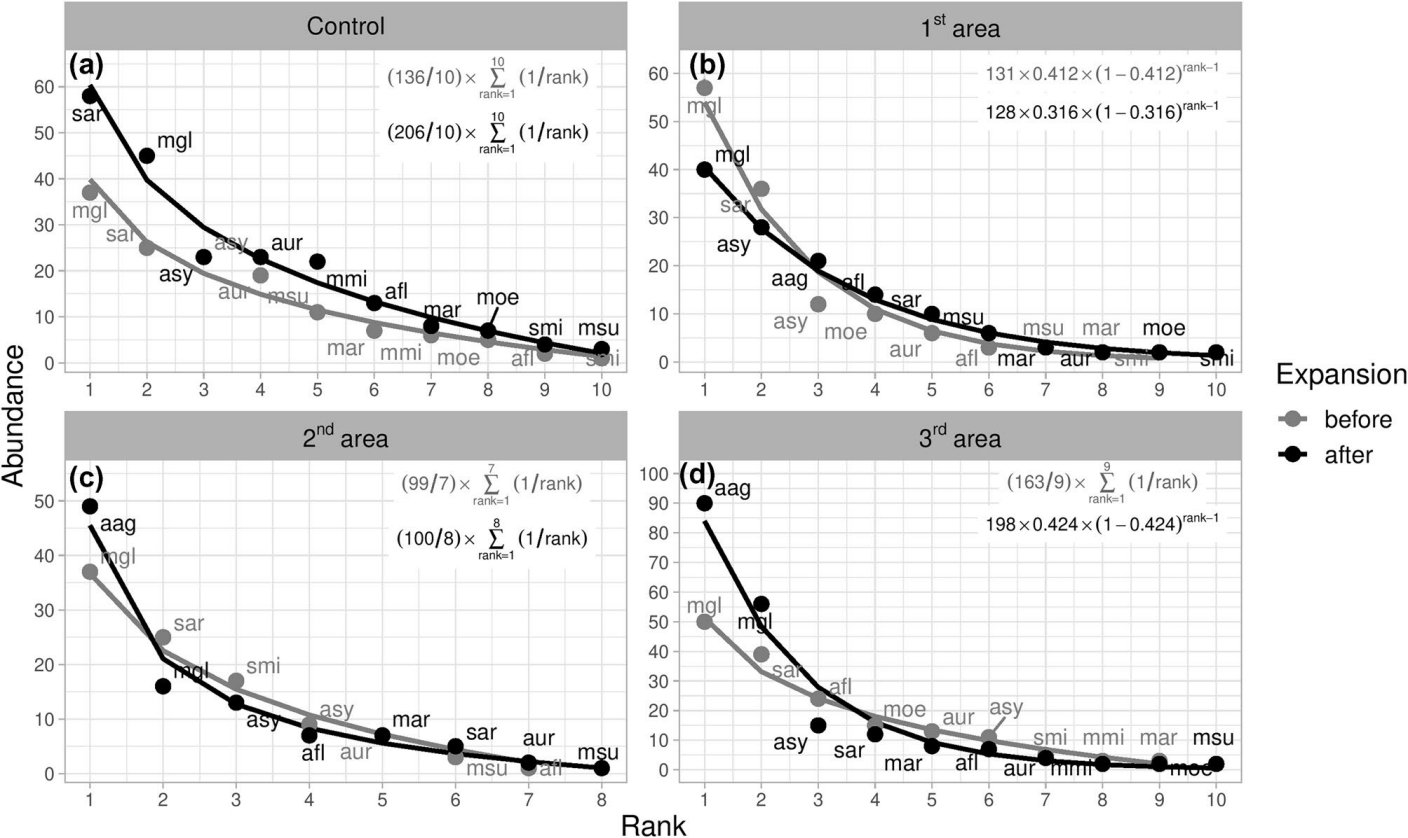
Rank abundance curves (RAC) of species in each study area before and after the striped field mouse’s expansion and in control sites during 1st and 2nd control periods. The lines represent the best-fit models of the abundance–rank relationship; mgl = *Myodes glareolus*, sar = *Sorex araneus*, asy = *Apodemus sylvaticus*, afl = *Apodemus flavicollis*, aur = *Apodemus uralensis*, mar = *Microtus arvalis*, moe = *Microtus oeconomus mehelyi*, smi = *Sorex minutus*, msu = *Microtus subterraneus*, mmi = *Microtus minutus*, csu = *Crocidura suaveolens.* Equations in the upper left-hand corner represent best-fit models of RACs.

The striped field mouse, after it had expanded its range, had a 100% frequency of occurrence at all of the 20 study sites. Prior analysis of community distribution types by RAC shows significant differences in rank change (χ^2^ = 9.08, *df* = 3, *p* = 0.028) when the striped field mouse became one of the most abundant species after its expansion and replaced the originally dominant bank vole (*Myodes glareolus*) (Fig. 2). In the first study area, the striped field mouse became the third-most abundant species. We also recorded a species re-ordering in the next position, which was previously held by the common shrew (*Sorex araneus*). The common shrew’s population fell after the striped field mouse’s expansion at all study areas except for the control sample, where it became the most abundant species in the control event. In contrast, the wood mouse shifted to a higher position in rank, but only became markedly more abundant in the first study area. Prior analyses showed no significant differences in other RAC aspects, such as species curve comparison, species richness comparisons, evenness comparisons, species gains and species losses (Supplementary S3).

**Figure 3:**
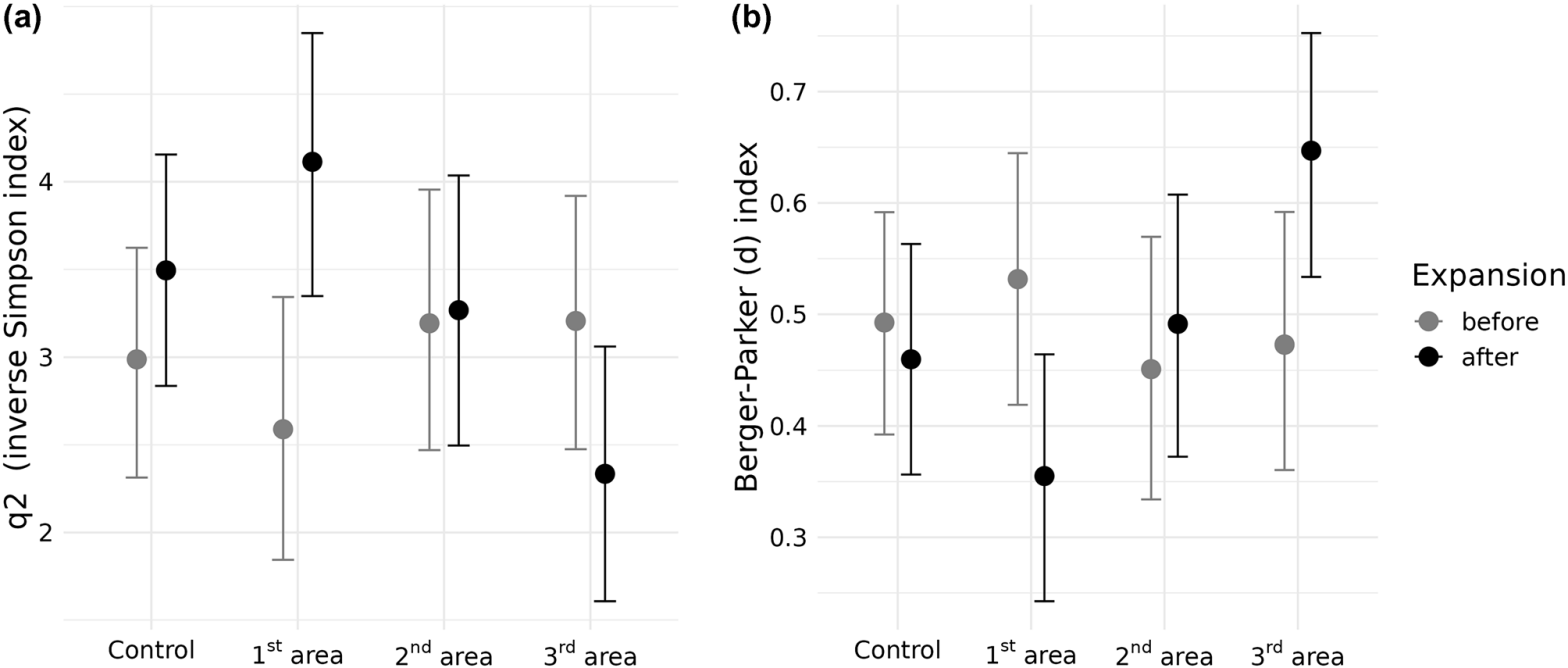
Expected values of posterior predictive distribution for the a) *q2* and b) Berger-Parker index, before and after the striped field mouse’s expansion into each study area. A lower Berger-Parker index indicates an increase in evenness. Whiskers represent 95% CI of mean estimate.

The results also show significant interaction between the study areas and community metrics caused by the striped field mouse’s expansion (for *q2*, Δelpd = − 4.4 with Δse = 2.2, and for the Berger–Parker index (*d*), Δelpd = 4 with Δse = 2.4). In the case of diversity (Fig. 3a) and evenness disturbance (Fig. 3b), contradictory effects were noticed between the first and third study areas. While the third study area showed a decrease after expansion, both metrics increased in the first study area. The second study area and control sample showed no significant differences (Supplementary S4).

**Figure 4:**
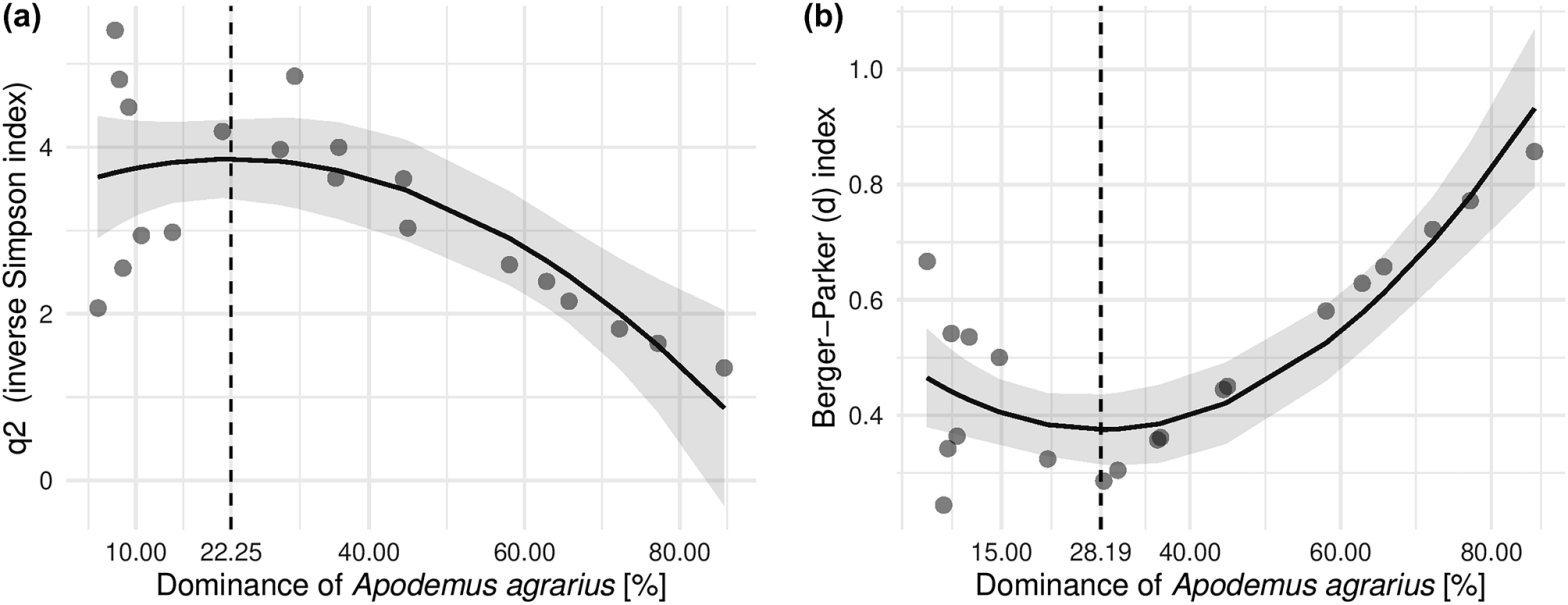
Second-degree polynomial relationship of the striped field mouse’s dominance for: (**a**) *q2* (inverse Simpson index), and (**b**) Berger–Parker index. Grey areas represent 95% CI. The dashed line shows the results from an optimised search of the minimum and maximum values of the functions. The dashed vertical line illustrates the break-even point for the negative impact of the striped field mouse’s dominance on diversity metrics of small mammal communities.

A comprehensive view of the relationship between the striped field mouse's dominance and community metrics is evident in a polynomial regression that shows the significant polynomial, unimodal relationship of *q2* (*F*_2,17_ = 10.05; *p* = 0.001) and the Berger-Parker index (*F*_2,17_ = 22.83; *p* < 0.001). Both models show an initial increase in community diversity (Fig. 4a) and evenness (Fig. 4b), with these metrics subsequently deteriorating as the striped field mouse’s dominance rises. Further exploration set a negative impact break-even point for striped field mouse’s dominance at 22% and 28%, respectively.

**Figure 5:**
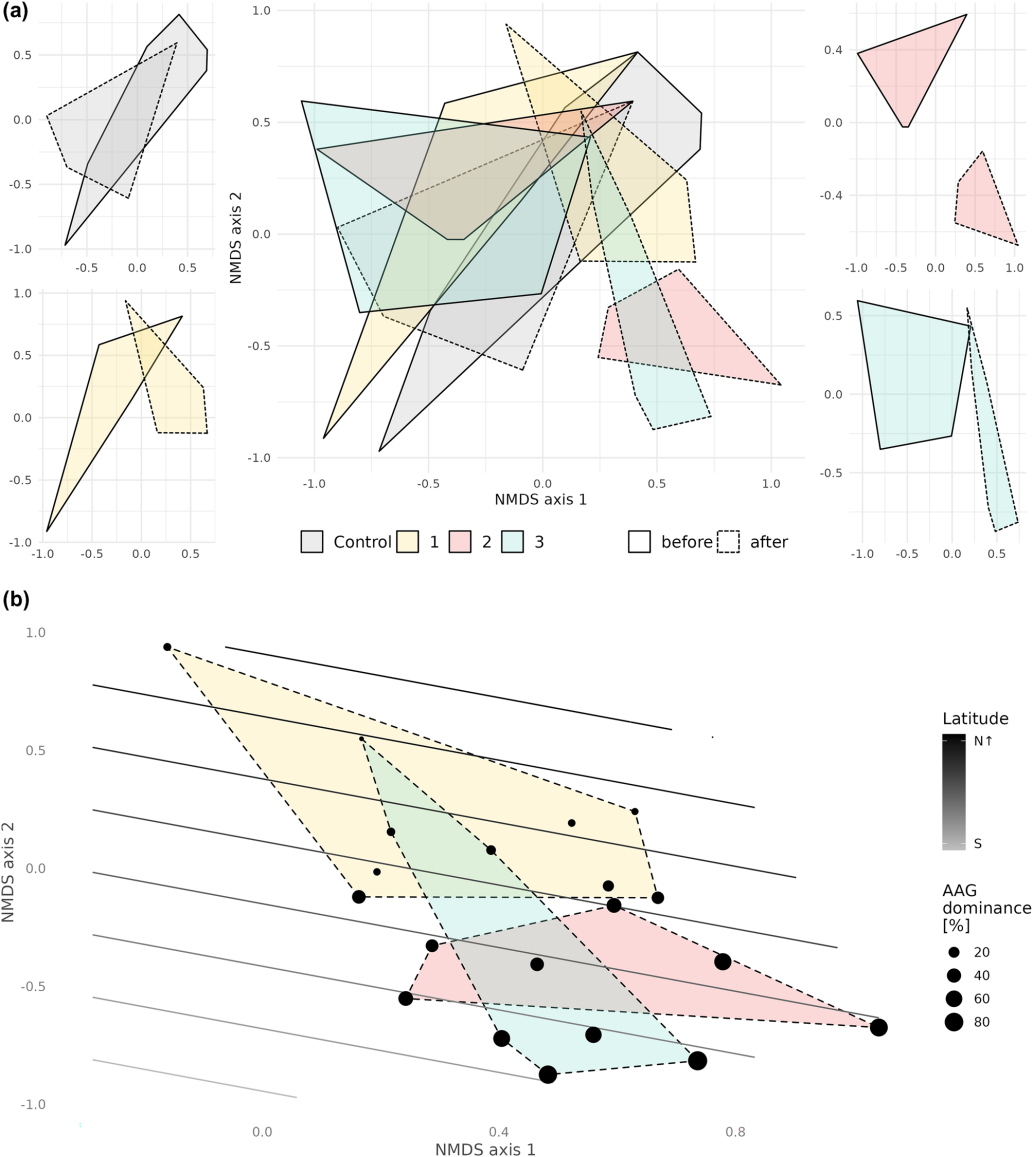
Non-metric multidimensional scaling (NMDS; stress = 0.194) of (**a**) small mammal community differences before and after the striped field mouse’s expansion (central figure = all study areas and control sites together, figures in corners = each study area and control separately) and (**b**) the effect of latitude and dominance on study sites and study area distribution; colours = study areas and control sites; solid line = study areas before striped field mouse expansion, dashed line = study areas after striped field mouse expansion; circle size = dominance of striped field mouse, colour of surface = changing latitude.

The similarity of small mammal communities was significantly affected by the striped field mouse’s expansion (MGLM: LRT = 57.45, *p* < 0.001), by the study area (MGLM: LRT = 102.59, *p* < 0.001) and by their interaction (MGLM: LRT = 62.60, *p* = 0.015) (Fig. 5a). While all three study areas indicate a high similarity before the expansion (MGLM: LRT = 52.72, *p* = 0.091), the post-expansion communities show similarity depending on the area (MGLM: LRT = 102.38, *p* < 0.001). Post hoc testing of data after expansionthen shows the differences between the control and the first area (MGLM: LRT = 45.27 *p*_adj_ = 0.015); control and second area (MGLM: LRT = 62.58, *p* = 0.003) and control and third area (MGLM: LTR = 44.07, *p* = 0.018).

The generalised additive model indicates a significant gradient in community composition changes (F_1.5, 9_ = 0.65, *p* = 0.026) and in the domination of the striped field mouse (F_2.9, 3.6_ = 7.06, *p* = 0.002) in the area towards the north-west (Fig. 5b).

## Discussion

### Establishment

Less than a decade after the first record of the striped field mouse’s expansion into south-western Slovakia^8^, the species had colonised all study sites. This finding corresponds with the strong ability of striped field mouse individuals to disperse and spread over a distance of 400–600 m, occasionally surpassing one kilometre^85,86^. Dudich^29^ estimated the speed of the species’ spread in southern Slovakia at between 25 and 30 kms over a 10–12 year period in the 1970s and 1980s, or three kilometres per year. The high degree of dispersion and success in colonisation are both characteristic of all invasive animal species, ranging from birds^87,88^ to larger mammals^89,90^, as well as invasive small mammals, such as the house mouse (*Mus m. domesticus*)^91^ and rat species (*Rattus rattus*, *Rattus axulans* and *Rattus norvegicus*)^92–94^. Ireland’s bank vole invasion began in the first decades of the nineteenth century and is still happening today, with the expansion rate estimated at 2.5 km per year^95^. The greater white-toothed shrew (*Crocidura russula*) has also expanded its range in Ireland since 2007 at an estimated rate of 0.5–14.1 km per year depending on the landscape^96^. However, detailed literature describing native small mammal range expansions is weak. Only Jareño et al.^17^ have recorded a rapidly expanded range (approx. 50,000 km^2^) for the common vole (*Microtus arvalis*) in Spain’s agricultural land over the past 40 years. Borrowing from invasion ecology theory, one stage in the invasion process is the individuals who are spreading having to establish a self-sustaining population within their new non-native range^6^. An established or naturalised non-native population may then grow in abundance and expand its geographic range. Both the occurrence of striped field mice in the diet of owls recorded from the Late and Early Holocene throughout the sixteenth century^36,38,97,98^, and changes in the range of occurrence during the last decades (see the introduction), describe a dynamic expansion and subsequent contraction of the striped field mouse's range or re-expansion of its distribution in Western and Central Europe in the past. However, no signs of this species’ range contracting in south-western Slovakia were observed until 2022. On the contrary, the species is still slowly penetrating the foothills and mountain valleys of the Carpathian Mountains, north of the study sites^58^.

### Impact on native population

Both Ambros et al.^8^ and Dudich^29^ expressed concerns about interspecific competition as the striped field mouse penetrated into the native populations of small mammals. In the newly colonised environment, the striped field mouse became either the most abundant or third-most abundant species. Our RAC results show the change in community level since the striped field mouse expanded its range and re-ordered the species, with a negative impact mainly on the bank vole and common shrew. The impact on bank voles was visible mainly in study areas with a higher striped field mouse dominance. Conversely, the abundance of bank voles rose where the striped field mouse was less dominant. While the abundance of the common shrew decreased in all study areas after the striped field mouse’s expansion, an abundance of wood mice increased only in the study area in which the striped field mouse was less dominant. The first indications of the negative impact of striped field mouse on the bank vole and common shrew had already been drawn five years after it expanded its range into south-western Slovakia^57^. Density-dependent competition is common in animal species^99^ and an important factor regulating population growth^100,101^. The growth rate of bank vole populations was negatively related to increasing densities of field voles (*Microtus agrestis*) in the increase phase of the vole cycle^102^, and, contrarily, its density grew twice in the absence of competitors^103^. The presence of the bank vole’s dominant competitors decreased females’ survival, their number, and the size of their territories^104,105^. Aggressiveness as a consequence of the competition between striped field mice and bank voles for available burrows has been directly observed^106^. Similarly, increasing total rodent numbers in the community negatively affected common shrew population growth rates^102^. Shrews had different habitat selection in the absence of competitors^107^, and Zub et al.^108^ indicated a possible competitive relationship between striped field mice and common shrews. Interspecific competition between shrews and rodents occurs primarily for space rather than for food, as herbivorous rodents and insectivorous shrews mostly subsist on different diets^109^. However, the high level of food plasticity among striped field mice^110–112^—an animal diet (mainly invertebrates) can constitute up to 30% of its diet^113,114^—can lead to higher competition as a result of food niche overlapping. Observations of a negative correlation between population sizes of sympatric small mammal species provided early indications that interspecific competition could have consequences on both population size and habitat use^115–117^. Our results on the community level again suggest concordance with invasion ecology, where becoming the dominant species in a colonised environment represents the next stage of invasion^7^ and competition and other interactions between non-native and native individuals are a part of a host of extrinsic forces determining whether new species are going to persist in the colonised environment^6^.

### Impact of the striped field mouse’s dominance at the community level

The variability of the striped field mouse’s dominance between the study areas led to a different impact on the researched community metrics. While low dominance increased diversity and evenness (higher *q2* values and lower Berger-Parker index), the high dominance of the striped field mouse showed a contradictory effect. No significant differences in the control sites indicated that these changes in community metrics are consequences of the striped field mouse’s expansion. Polynomial models confirmed an initial positive impact on community diversity (higher *q2* values) and evenness (lower Berger-Parker index), with low striped field mouse dominance alongside a subsequently rapid decrease in diversity (lower *q2* values) and evenness (higher Berger-Parker index) as dominance rose. On average, the break-even point of the negative impact on native small mammal communities was at 25% of the striped field mouse’s dominance. Having a negative impact on the native community and its diversity as a whole is one of the main criteria of becoming an invasive species^4,5^, which has been well documented for a wide spectrum of invaders, ranging from plants^118,119^, non-mammalian vertebrates^120–122^ and mammals^123^, to small mammals on islands^124,125^—though it is less documented for small mammals on continents^91,126^. Most of these studies have mainly measured the impact on species richness. Bradley et al.^9^ documented that the negative effect of an invader´s abundance is significantly stronger for evenness and diversity than for richness, which as a conservative measure of community-level changes requires species extinctions or additions to register change. Bradley et al.^9^ again reviewed that as invaders become more abundant community-level impacts are clearly negative; these statements fully correspond with our results.

In invasion ecology, non-native species can reduce variability between communities over time in a process of homogenisation where a widespread species becomes dominant and replaces other native, often rare species^127,128^. In our results, communities of small mammals were already showing high levels of similarity before the striped field mouse’s expansion, which was expected due to the similarities between the study sites. Our analysis, thus, suggests that the differences between recent (post-expansion) communities are a consequence of expansion and different levels of the striped field mouse’s dominance across the study areas.

### Dominance as a dispersal direction indicator?

Population spread is a consequence of dispersal and population growth. Dispersal moves individuals through space (some of these individuals move into uncolonised territory), and population growth causes the low-density edge populations to increase in density over time^129^. The invasion front is characterised by lower densities relative to those behind it^130^. This density gradient moves through space and is persistent over time, so long as the population is spreading^129^. In a study from 2016, we suggested a north to north-east dispersal direction of the striped field mouse’s expansion^57^. Based on the decrease in the striped field mouse’s dominance, however, our current results indicate a north-west expansion direction. Therefore, we assume that the first studied area was colonised after the other two. This hypothesis is supported by Stanko^131^, who in many cases documented the gradual increase in the dominance of the striped field mouse in colonised communities, and by new occurrences of the striped field mouse found farther north of the first study area^58^. However, determining the spread’s direction was not the primary goal of this study, and confirmation of this hypothesis would require a different, extended type of study design.

### “Invasion” of a native species?

Transport—whether biotic, abiotic or human-mediated—to novel areas is considered the first fundamental stage of the invasion process^6^. The origin of the striped field mouse population in the study region has not yet been clarified and based on known indications of its life history (see Introduction) we recently considered it a native species expanding its range. However, expansions of native organisms into adjacent communities can emulate the functional and structural changes, as well as the impact on biodiversity, associated with invasions by alien species^10^. Tong et al.^19^ argued that expanding native species can move together with their associated organisms and, thus, do not benefit from enemy release like invasive alien species, so the effects of native species’ range expansion on local communities are typically more neutral and milder than alien invasions. Colautti and MacIsaac^7^ proposed that transport and other invasion stages as an introduction or establishment could be used to model the local spread of more types of potential colonisers, and might be available from a regional species pool including native species. Several studies have suggested that processes affecting local spread and establishment in novel areas may be independent of species origin^21–23,132^. Indeed, nonindigenous species are actually only nonindigenous populations of species; the same “species” that are nonindigenous, naturalised, or invasive in one area are native somewhere else^7^. Our results suggest several identical stages of invasions and invasion success for the striped field mouse’s expansion in south-western Slovakia. The species was able to establish a self-sustaining population, expand its range, become the dominant unit and, overall, affect the native population and communities. The results are, thus, in accordance with both Thompson et al.^132^, who argued that the attributes of invasive aliens are shared by native species and not unique, and Nackley et al.^10^, who argued that, in many respects, expanding native species can be functionally indistinguishable from invading alien species.

In conclusion, over the several years since the striped field mouse expanded into south-western Slovakia it has successfully colonised, and continues to persist in, all study sites. Although neither the causes nor the origins of its current expansion have yet been elucidated, the dominance of the species has nevertheless had a determinedly significant impact on the native community, specifically: (i) the rise of its domination led to a re-ordering of other species, and (ii) the initial positive effect of the expansion on the community metrics, when the striped field mouse exercised low dominance, turned markedly negative after crossing the 25% dominance threshold. The experience gained from a live history of striped field mice, and both the consequences and risks from similar invasions, oblige us to focus future studies on the current range of occurrence and possible retreat from colonised territory, to identify the origin of expanding populations, and to analyse potential threats of this epidemiologically major species.

## Supplementary Information

Supplementary Information 1.

Supplementary Information 2.

## Data Availability

All data generated or analysed during this study are included in this published article [and its supplementary information files].
