# Author Correction: The impact of the striped field mouse’s range expansion on communities of native small mammals

**DOI:** 10.1038/s41598-023-36731-y

**Published:** 2023-06-20

**Authors:** Filip Tulis, Michal Ševčík, Radoslava Jánošíková, Ivan Baláž, Michal Ambros, Lucia Zvaríková, Gyözö Horváth

**Affiliations:** 1 Department of Ecology and Environmental Science, Faculty of Natural Sciences and Informatics, Constantine the Philosopher University in Nitra, Tr. A. Hlinku 1, SK-949 74 Nitra, Slovak Republic; 2 State Nature Conservancy of SR, Administration of Protected Landscape Area Ponitrie, Samova 3, SK-949 01 Nitra, Slovak Republic; 3 Department of Ecology, Institute of Biology, Faculty of Sciences, University of Pécs, Ifjúság Útja 6, H-7624 Pécs, Hungary

Correction to: *Scientific Reports* https://doi.org/10.1038/s41598-022-26919-z, published online 14 January 2023

The original version of this Article contained errors in the names of authors Filip Tulis, Michal Ševčík, Radoslava Jánošíková, Ivan Baláž, Michal Ambros, Lucia Zvaríková and Gyözö Horváth, which were incorrectly given as Tulis Filip, Ševčík Michal, Jánošíková Radoslava, Baláž Ivan, Ambros Michal, Zvaríková Lucia and Horváth Gyözö.

The original Article has been corrected.

