# Supplementary material for: The impact of the striped field mouse’s range expansion on communities of native small mammals
Source: Sci Rep. 2023 Jan 14;13:753. doi: 10.1038/s41598-022-26919-z (PMC9840622; doi:10.1038/s41598-022-26919-z)
Supplement: Supplementary file 2 — Supplementary Information 2. [file 41598_2022_26919_MOESM2_ESM.docx]

**Supplementary**

**Supplementary 1:** Recorded small mammals at given localities, study area, study site, year – before, after expansion and before expansion – control event.

*See Excel attachment*

**Supplementary 2:** Saturated species diversity from Hill numbers (*q0* = richness, *q1* = exponential Shannon index, *q2* = inverse Simpson index) for small mammal communities studied before and after expansion of the striped field mouse’s range for study sites and all data together


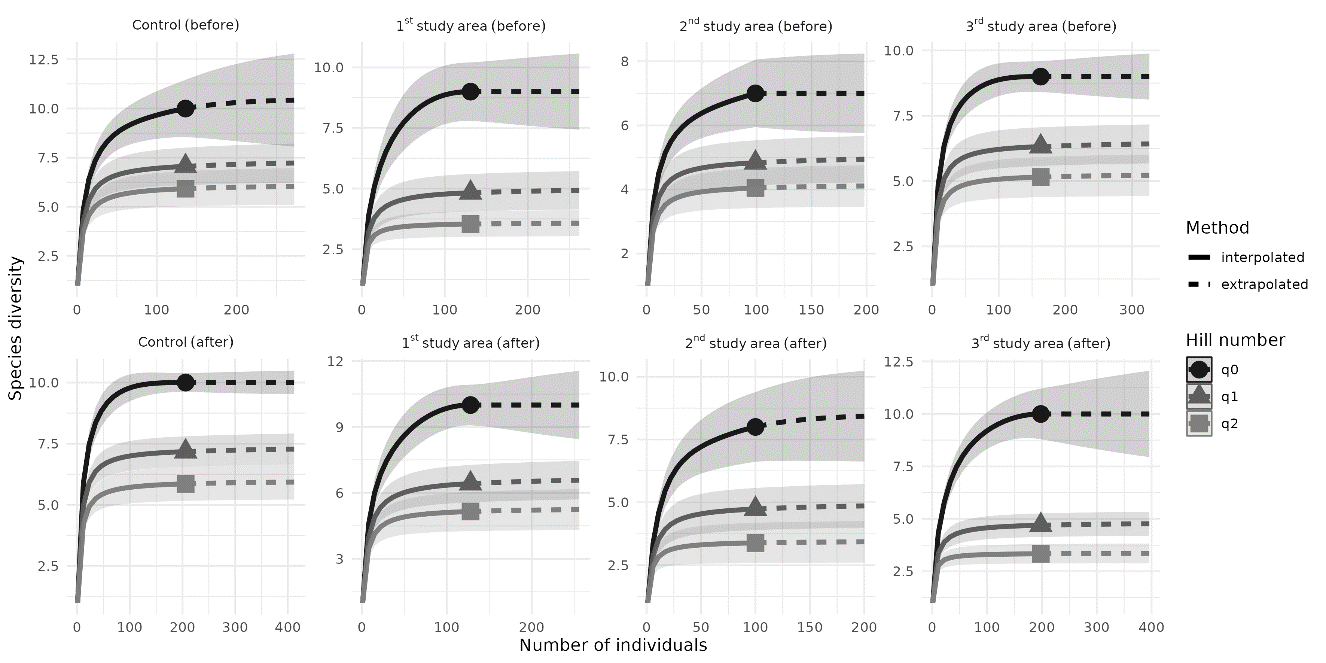


**Supplementary 3:** Results of Kruskal-Wallis test of community dynamics parameters between localities

| Parameter | Test statistics (χ^2^) | Degrees of freedom | *p*-values |
| --- | --- | --- | --- |
| Richness change | 1.0863 | 2 | 0.58092 |
| Evenness change | 3.11701 | 2 | 0.21045 |
| Rank change | 8.66149 | 2 | 0.013158 |
| Gains | 0.701894 | 2 | 0.70402 |
| Losses | 5.78065 | 2 | 0.055558 |
| Curve change | 1.32449 | 2 | 0.51569 |

**Supplementary 4:** Results of post hoc hypothesis testing of differences before and after expansion per locality

| Hypothesis | Parameter | Est. difference (95% CI) |
| --- | --- | --- |
| Before Control – After Control = 0 | *q2* | -0.52 (-1.34; 0.33) |
|  | Berger-Parker (*d*) | 0.03 (-0.10; 0.17) |
| Before 1^st^ – After 1^st^ = 0 | *q2* | -1.52 (-2.53; -0.48) |
|  | Berger-Parker (*d*) | 0.18 (0.03; 0.33) |
| Before 2^nd^ – After 2^nd^ = 0 | *q2* | -0.07 (-1.10; 0.99) |
|  | Berger-Parker (*d*) | -0.04 (-0.21; 0.13) |
| Before 3^rd^ – After 3^rd^ = 0 | *q2* | 0.88 (-0.08; 1.86) |
|  | Berger-Parker (*d*) | -0.18 (-0.32; 0.02) |
